# Targeting TXNIP in endothelial progenitors mitigates IL-8-induced neutrophil recruitment under metabolic stress

**DOI:** 10.1186/s13287-024-03850-w

**Published:** 2024-07-29

**Authors:** Julia Jolibois, Alison Domingues, Divina El Hamaoui, Raphaël Awaida, Mathilde Berger-de-Gaillardo, Daniel Guérin, David M Smadja, Perrine Marquet-DeRougé, Isabelle Margaill, Elisa Rossi, Valérie Nivet-Antoine

**Affiliations:** 1 Université Paris Cité, INSERM, Innovations thérapeutiques en hémostase, Paris, F-75006 France; 2 Laboratoire de Biochimie générale, AP-HP, Hôpital Necker Enfants Malades, Paris, F-75015 France; 3 Laboratoire d’Hématologie, AP-HP, Hôpital Européen Georges Pompidou, Paris, F-75015 France

**Keywords:** Endothelial progenitors, Metabolic stress, Age-related comorbidities, TXNIP, Cytokine secretion, Neutrophils

## Abstract

**Background:**

This study explores the potential role of Thioredoxin-interacting protein (TXNIP) silencing in endothelial colony-forming cells (ECFCs) within the scope of age-related comorbidities and impaired vascular repair. We aim to elucidate the effects of TXNIP silencing on vasculogenic properties, paracrine secretion, and neutrophil recruitment under conditions of metabolic stress.

**Methods:**

ECFCs, isolated from human blood cord, were transfected with TXNIP siRNA and exposed to a high glucose and β-hydroxybutyrate (BHB) medium to simulate metabolic stress. We evaluated the effects of TXNIP silencing on ECFCs’ functional and secretory responses under these conditions. Assessments included analyses of gene and protein expression profiles, vasculogenic properties, cytokine secretion and neutrophil recruitment both in vitro and in vivo. The in vivo effects were examined using a murine model of hindlimb ischemia to observe the physiological relevance of TXNIP modulation under metabolic disorders.

**Results:**

TXNIP silencing did not mitigate the adverse effects on cell recruitment, vasculogenic properties, or senescence induced by metabolic stress in ECFCs. However, it significantly reduced IL-8 secretion and consequent neutrophil recruitment under these conditions. In a mouse model of hindlimb ischemia, endothelial deletion of TXNIP reduced MIP-2 secretion and prevented increased neutrophil recruitment induced by age-related comorbidities.

**Conclusions:**

Our findings suggest that targeting TXNIP in ECFCs may alleviate ischemic complications exacerbated by metabolic stress, offering potential clinical benefits for patients suffering from age-related comorbidities.

**Supplementary Information:**

The online version contains supplementary material available at 10.1186/s13287-024-03850-w.

## Introduction

Progenitor cells are crucial for managing postischemic therapies, especially in hindlimb ischemia diseases [1, 2]. Among these, endothelial progenitor cells (EPCs) are particularly promising, as they promote endothelial repair and angiogenesis through physical integration into vascular walls and paracrine activities [3, 4]. Since their discovery, numerous studies have highlighted the role of EPCs in enhancing vascularization across species, from rodents [5, 6] to humans [7, 8]. Originating from bone marrow, EPCs are mobilized into the peripheral circulation in response to chemokines such as vascular endothelial growth factor (VEGF) and stromal-derived factor-1 (SDF-1) and migrate to injury sites, where they support vascular network development and mature into endothelial cells [9–11]. Alternatively, EPCs can control angiogenesis through paracrine signaling *via* the secretion of cytokines and/or growth factors, producing an angiogenic microenvironment. This stimulates the proliferation and migration of vessel-forming cells, which play a crucial role in maintaining vascular integrity [3, 12].

In postischemic disease progression, initiating a proangiogenic response is therapeutically challenging, particularly in patients with age-related comorbidities. Revascularization and maintenance of vascular integrity depend largely on the functional capacity and number of endothelial cells and their progenitors. Despite the common assumption of a decrease in these factors with age and comorbidities [13, 14], clinical studies, such as those conducted by Heiss et al. [15], indicate that while the function of EPCs may be compromised in elderly patients, their numbers do not necessarily decrease, suggesting a complex interaction between aging and EPC functionality. Moreover, age-related comorbidities create a harmful environment that can adversely affect the secretion profile of EPCs, further impairing vascular repair [16].

Among EPCs, two distinct subtypes isolated in culture have been described: myeloid angiogenic cells (MACs) and endothelial colony-forming cells (ECFCs). ECFCs display an unequivocal endothelial phenotype and possess an inherent ability to promote vasculogenesis [17]. These cells have a robust intrinsic angiogenic capacity and can release paracrine factors to repair injured endothelium [18, 19].

Thioredoxin-interacting protein (TXNIP) is a multifunctional protein involved in the regulation of cellular homeostasis. It is a regulator of metabolism [20, 21] and modulator of angiogenic [22, 23] and inflammatory [24] responses. It may also influence the quiescence and migration of hematopoietic stem cells by regulating intracellular oxidative stress [25, 26]. Reducing endothelial TXNIP expression helps protect against arterial damage caused by age-related comorbidities [27]. Recently, we reported that endothelial TXNIP targeting in the context of age-related comorbidities is essential for restoring cell sensitivity to angiogenic factors and improving postischemic revascularization [23].

Our hypothesis is that the observed benefits of endothelial TXNIP deletion in the context of age-related comorbidities may arise from protecting the vascular system and potentially influencing endothelial progenitors. This study aimed to delineate for the first time how TXNIP-silenced progenitors such as ECFCs contribute to the beneficial effects of endothelial TXNIP deletion on angiogenesis and postischemic revascularization in age-related comorbidities. To assess the role of ECFCs in this process, we designed a metabolic stress model reflecting age-related comorbidities observed in vivo. We targeted TXNIP in ECFCs and then investigated how TXNIP silencing in this context affects their recruitment, vasculogenic properties, senescence, secretion patterns, and neutrophil recruitment.

## Methods

### Cell isolation and culture

Umbilical cord blood for the study was obtained from AP-HP, Hôpital Saint-Louis, Unité de Thérapie Cellulaire, and CRB-Banque de Sang de Cordon in Paris, France, after normal full-term deliveries with written informed consent from the mothers. Endothelial colony-forming cells (ECFCs) were isolated from these samples under authorization AC-2016-2759. This authorization is not related to a specific project as part of the CRB and therefore does not have a study title or an ethics committee. Cord-blood ECFCs were isolated from the adherent mononuclear cell fraction using Ficoll density centrifugation as previously described [17]. Briefly, the cells were cultured in EGM-2 MV (Lonza, USA) supplemented with 10% fetal bovine serum. ECFCs appear in culture after 7–20 days of plating and form highly proliferative colonies microscopically visible. Then the cells were trypsinized and expanded on fibronectin-coated plates (1 µg/cm2; Merck, Germany) in 6-well plates. ECFCs were used between passages 4 and 7. The ECFCs were cultured at 37 °C and 5% CO2.

### Metabolic stress induction and cell transfection

TXNIP-specific siRNA (VDUP1 siRNA (h); sc-44,943, Santa Cruz Biotechnology, USA) was used to silence human TXNIP (siTXNIP). Briefly, 10 µM siRNA was mixed with DharmaFECT reagent (T-2002-03, Dharmacon, USA) to obtain transfection complexes, which were added to cells in 6-well plates containing EGM-2MV (without antibiotics). Cells transfected with scrambled siRNA (control siRNA-A; sc-37,007; Santa Cruz Biotechnology, USA) were used as a control (siSCR). One day later, EGM-2MV supplemented with 25 mM glucose (Sigma, USA) and 10 mM β-hydroxybutyrate (BHB) (Sigma, USA) (GB) or control medium (CT) was added for 24 h to induce metabolic stress. The experimental design results in 4 distinct groups: control transfection without metabolic stress (siSCR-CT), control transfection with metabolic stress (siSCR-GB), TXNIP silencing without metabolic stress (siTXNIP-CT), and TXNIP silencing with metabolic stress (siTXNIP-GB).

### Quantitative real-time polymerase chain reaction (RTqPCR)

Cultured cells were lysed in RLT buffer, and RNA was extracted with an RNeasy Microkit (Qiagen, Germany). The RNA concentration and purity were measured using a NanoDrop One/One (Thermo Fisher Scientific, USA). Then, reverse transcription of cDNA was performed using a QuantiTect Reverse Transcription Kit (Qiagen, Germany). Both procedures were performed according to the manufacturer’s specifications. A CFX384 Touch Real-Time PCR Detection System (Bio-Rad, USA) was used for real-time PCR amplification. QuantiTect SYBR Green PCR kits and QuantiTect primer assays were used to quantify TXNIP (Hs_TXNIP_1_SG QuantiTect Primer Assay, Qiagen, Germany). All the reactions were performed in triplicate in a final volume of 20 µl according to the manufacturer’s instructions. Ribosomal protein L13 (RPL13) (Hs_MRPL13_1 SG QuantiTect Primer Assay, Qiagen, Germany) was used as the housekeeping gene after the validation step. The data were analyzed by the 2^−ΔΔ Ct^ method, as described by Livak and Schmittgen [28] and are expressed as the “mRNA fold change”.

### Western blot

ECFC lysates were prepared with cell extraction buffer (Invitrogen, USA) supplemented with Complete and PhoSstop (Roche, Switzerland). Protein concentrations were determined using a Pierce BCA Protein Assay Kit (Thermo Fisher Scientific, USA). Denaturing NuPAGE and Western blotting were conducted using precast 4-12% Bis-Tris polyacrylamide gels (Invitrogen, USA) for protein separation. After separation, the proteins were transferred to nitrocellulose membranes using a dry system (Iblot Gel Transfer System, Invitrogen, USA). The membranes were blocked with 5% skimmed milk in TBST, followed by incubation with an anti-TXNIP antibody (1/500, Invitrogen, USA) overnight at 4 °C and for 45 min at room temperature with fluorescent-labeled secondary antibodies [Dylight 800-conjugated secondary antibody (1:20,000, Thermo Fisher Scientific, USA)]. Fluorescent immunoblot images were acquired using an Odyssey scanner (LI-COR Biosciences, USA) and quantified using ImageJ software.

### Chemotactic migration assay

Twenty-four-well Transwell PET membranes with 8-µm-diameter pores (Corning, USA) were coated with 10 µg/mL fibronectin at 37 °C for 30 min. Subsequently, 100 µl containing 2 × 10^4^ ECFCs/well were loaded into the upper part of the chamber with either CT or GB medium, In the lower chamber, 600 µL of CT medium or GB medium with or without 50 ng/mL VEGF (R&D Systems, USA) was added. The same experiment was performed by replacing VEGF with another chemoattractant, 100 ng/mL SDF-1 (R&D Systems, USA). Migration was allowed to progress for 4 h at 37 °C. The membranes were then fixed in 4% PFA (Thermo Fisher Scientific, USA), and the cells remaining on the upper surface of the membrane were removed with a cotton swab. Mounting was achieved with Vectashield mounting medium containing DAPI (H-1200, Vector Labs, USA), and the membranes were imaged with an epifluorescence microscope (x100 magnification, Zeiss AXIO, Germany). For each membrane, the numbers of cells in four separate fields were quantified with ImageJ software. These assays were conducted in duplicate with six independent experiments using different cell preparations.

### In vitro Matrigel^®^ tube formation assay

Matrigel™ Matrix Growth Factor Reduced (Corning, USA) was prepared at 4 °C in 48-well plates on crushed ice. Gel polymerization was achieved by incubating the plate at 37 °C for 1 h. A total of 90 000 transfected ECFCs/well were added to 500 µL of CT or GB medium. The formation of pseudotubes was observed after 4, 7 and 20 h of incubation at 37 °C, and images were taken with a microscope (40x magnification). Quantification of the total length of the pseudotubes formed and the number of junctions was performed using Angiogenesis Analyzer plugin on ImageJ software. These assays were conducted in duplicate with five independent experiments using different cell preparations.

### Wound healing assay

All angiogenesis assays were performed according to consensus guidelines [29].

Transfected ECFCs were seeded at 300 000 cells/well in 24-well plates with or without metabolic stress (CT or GB). The next day, a “wound” was made at T0. Images were taken at 0, 2, 4, 7 and 24 h after wounding (x40 magnification). These images were analyzed with ImageJ software. The percentage of wound closure was calculated relative to the area of the initial wound. These assays were conducted in duplicate with seven independent experiments using different cell preparations.

### Senescence assay

ECFCs were seeded in 24-well plates at 20,000 cells per well. Senescence was quantified *via* colorimetric detection of senescence-associated ß-galactosidase (SA-β-gal) using a Senescence Detection Kit (Promega, Germany) according to the manufacturer’s instructions. SA-β-gal-positive cells were counted in six randomly selected microscopic fields (×200 magnification). The results are expressed as the percentage of total cells. These assays were conducted in duplicate with five independent experiments using different cell preparations.

### Soluble factors secreted by ECFCs

Cultured transfected cells (siSCR and siTXNIP) were grown for 24 h in CT or GB media. Subsequently, the conditioned media were harvested and stored at − 80 °C. Simultaneously, the cells were counted. Upon thawing, the conditioned media were centrifuged at 1500 × g for 6 minutes. The concentrations of cytokines were determined using a Luminex Human Discovery Assay (R&D Systems, USA) according to the manufacturer’s instructions. Briefly, conditioned media were incubated with fluorescent beads conjugated to a panel of antibodies, each specific for a distinct factor. The hybridization of a secondary biotinylated antibody allowed for precise quantification of soluble factors after the addition of streptavidin–phycoerythrin. The analysis was conducted using Bioplex Manager Software (Bio-Rad, USA). All results are expressed in ng- or pg/10^6^ cells. These assays were conducted in duplicate with six independent experiments using different cell preparations.

### In vitro neutrophil migration assay

Blood samples collected on EDTA were obtained from healthy volunteers from Etablissement Français du Sang (EFS, convention n°13/EFS/064). Neutrophils were isolated with a MACSxpress Neutrophil Isolation Kit (Miltenyi Biotech, Germany) according to the manufacturer’s instructions. Twenty-four-well Transwell PET membranes with 5 μm pore diameters (Corning, USA) were blocked with EBM-2 0.1% BSA (Sigma, USA) at 37 °C for 30 min. Then, 1 × 10^5^ neutrophils/well were loaded in EGM-2 MV into the upper part of the chamber. The lower part of the chamber was filled with conditioned media. The cells were allowed to migrate for 2 h at 37 °C. Subsequently, the medium in the lower part of the chamber was collected, and the neutrophils were labeled with CD15^+^/CD16^+^ (Miltenyi Biotech, Germany) and quantified using flow cytometry (BD LSRFortessa, Beckton Dickinson, USA). The results are expressed as the number of neutrophils that migrated. These assays were conducted in duplicate with four independent experiments using different cell preparations.

### Murine model of age-related comorbidities

Ethical approval for all animal experiments was obtained from the Ethics Committee on Animal Resources at Paris Descartes University, and the study was registered with the French Ministry of Higher Education and Research under number #4920 in 2016 (“Ischémie du membre inférieur chez des souris invalidées (Ec-TXNIP) lors du vieillissement”). This approval and registration were conducted in accordance with Directive 2010/63/EU and ARRIVE guidelines (see ARRIVE checklist in Additional file 3). In this protocol, the number of animals was determined based on our extensive experience on the models. For this study, samples from mice were collected as part of procedures that have been previously published, contributing to the limitation of animal use [23]. Mice were maintained in the same room from animal facility (Plateforme Pharmanima) of the UAR3612 CNRS-US25 INSERM Faculté de Pharmacie, Université Paris Cité, in an environment with regulated temperature and humidity, followed by a 12-hour light/dark cycle, and had continuous access to water and food. At the age of six months, both male and female mice were divided into two dietary groups for a duration of 3 months: one group received a standard control diet (M20, Special Diets Services, Witham, UK), while the other was given a high-fat high-protein low-carbohydrate (HFHPLC) diet (U8954, Scientific Animal Food and Engineering, Augy, France), allowing the development of age-related comorbidities in a murine model [30]. The number of animals was chosen based on the validation of the diet on glucose tolerance test. To account for an exclusion rate due to diet inefficacy, we have increased the size of HFHPLC diet group.

### Endothelial TXNIP deletion in mice

To generate a cell-specific TXNIP knockout mouse, the Cre-LoxP system was used [31]. TXNIP^fl.^/^fl.^ mice (B6, 129 S-Txniptm1Rlee/J, 16,847) purchased from the Jackson Laboratory (Bar Harbor, ME, USA) were crossed with Cre recombinase transgenic mice under the control of the VE-cadherin 5 promoter [B6. Cg-Tg(Cdh5-Cre)7Mlia/J, 6137; Jackson Laboratory], which induces recombination in endothelial cells and a portion of hematopoietic cells such as endothelial progenitors [31]. Specific TXNIP knockout mice (TXNIP^fl.^/^fl.^cdh5^cre^) were obtained, and control Cre-cadherin littermate mice were used as controls. Genotyping was performed as recommended by the Jackson Laboratory. Immunofluorescence labeling and quantification validated the model, as described previously [27]. Male and female mice were randomized at the age of 6 months and divided into four groups according to their diet: (i) cre-cadherin littermate control mice fed a standard control diet (control with control diet); (ii) cre-cadherin littermate control mice fed an HFHPLC diet (control with HFHPLC diet); (iii) TXNIP^fl.^/^fl.^cdh5^cre^ fed a standard control diet (TXNIP^fl.^/^fl.^cdh5^cre^ with control diet); and (iv) TXNIP^fl.^/^fl.^cdh5^cre^ fed an HFHPLC diet (TXNIP^fl.^/^fl.^cdh5^cre^ with HFHPLC diet).

### Murine model of hindlimb ischemia

#### Ethical approval

for all animal experiments was obtained from the Ethics Committee on Animal Resources at Paris Descartes University, and the study was registered with the French Ministry of Higher Education and Research under number #4920 in 2016 (“Ischémie du membre inférieur chez des souris invalidées (Ec-TXNIP) lors du vieillissement”) in accordance with Directive 2010/63/EU and ARRIVE guidelines (see ARRIVE checklist in Additional file 3). Every mouse in each group were randomly subjected to surgery at the age of 9 months as previously described, ensuring a uniform distribution to minimize group bias [23]. Briefly, mice were anesthetized with isoflurane and received analgesia with buprenorphine. After incision of the skin, the left common femoral artery and vein were double ligated, followed by resection of the ligatured zone and its collaterals. The mouse is then sutured and placed in a temperature-controlled recovery cage before being returned to its cage mates. A monitoring score is conducted until euthanasia of the animals. The mice were maintained for 21 days after surgery and euthanized by overdose of anesthetic and exsanguination. The overdose of anesthetic was administered using 5% isoflurane, and exsanguination was performed via intracardiac blood collection. Confirmation of euthanasia was subsequently conducted by harvesting various organs, including the brain.

### Blood lactate, β-hydroxybutyrate and MIP-2 quantification

The concentrations of serum or conditioned medium lactate and β-hydroxybutyrate were measured on an Alinity Analyzer using enzymatic methods (Abbott, France). The concentration of macrophage inflammatory protein 2 (MIP-2) in mouse serum was measured by enzyme-linked immunosorbent assay (ELISA) with a Quantikine ELISA kit (Biotechne, R&D Systems, USA) following the manufacturer’s instructions.

### Immunofluorescence labeling of calf muscle

Calf muscle samples from ischemic legs were fresh-frozen in TissueTek optimum cutting temperature compound medium and stored at − 80 °C. Cryostat sections were prepared and fixed (4% paraformaldehyde). After blocking, the sections were incubated with a primary antibody against myeloperoxidase (MPO) (rabbit pAb anti-MPO; Agilent). Labeling was visualized with Alexa Fluor 555-conjugated goat anti-rabbit antibodies (Thermo Fisher Scientific, USA). Nuclei were counterstained with DAPI. Negative controls with a nonimmune IgG isotype did not yield any detectable labeling. Images were acquired on a Leica Thunder Imager (x40 magnification). At least three sections of each muscle were recorded. The average neutrophil count was determined by the number of MPO-positive cells, expressed per unit of area analyzed and quantified using NIH ImageJ software. Quantification was independently carried out by two operators in a blind manner, and the results shown are the means of these two independent assessments.

### Statistical analysis

All analyses were performed with Prism software 10 (GraphPad). Statistical analysis was performed by 2-way ANOVA followed by post hoc comparison with Fisher’s LSD test or the Sidak test when studying 2 factors: the effect of metabolic stress and TXNIP silencing. Paired t tests were used for comparisons of 2 paired groups when the data were normally distributed, or Wilcoxon signed-rank tests were used for comparisons. An unpaired t test with Welch’s correction was used for comparisons of 2 independent groups. A p value < 0.05 was considered to indicate statistical significance. Aberrant values were identified with the ROUT test and excluded from analysis.

## Results

### In vitro metabolic stress accurately simulates the age-related comorbidities observed in vivo

**Fig. 1:**
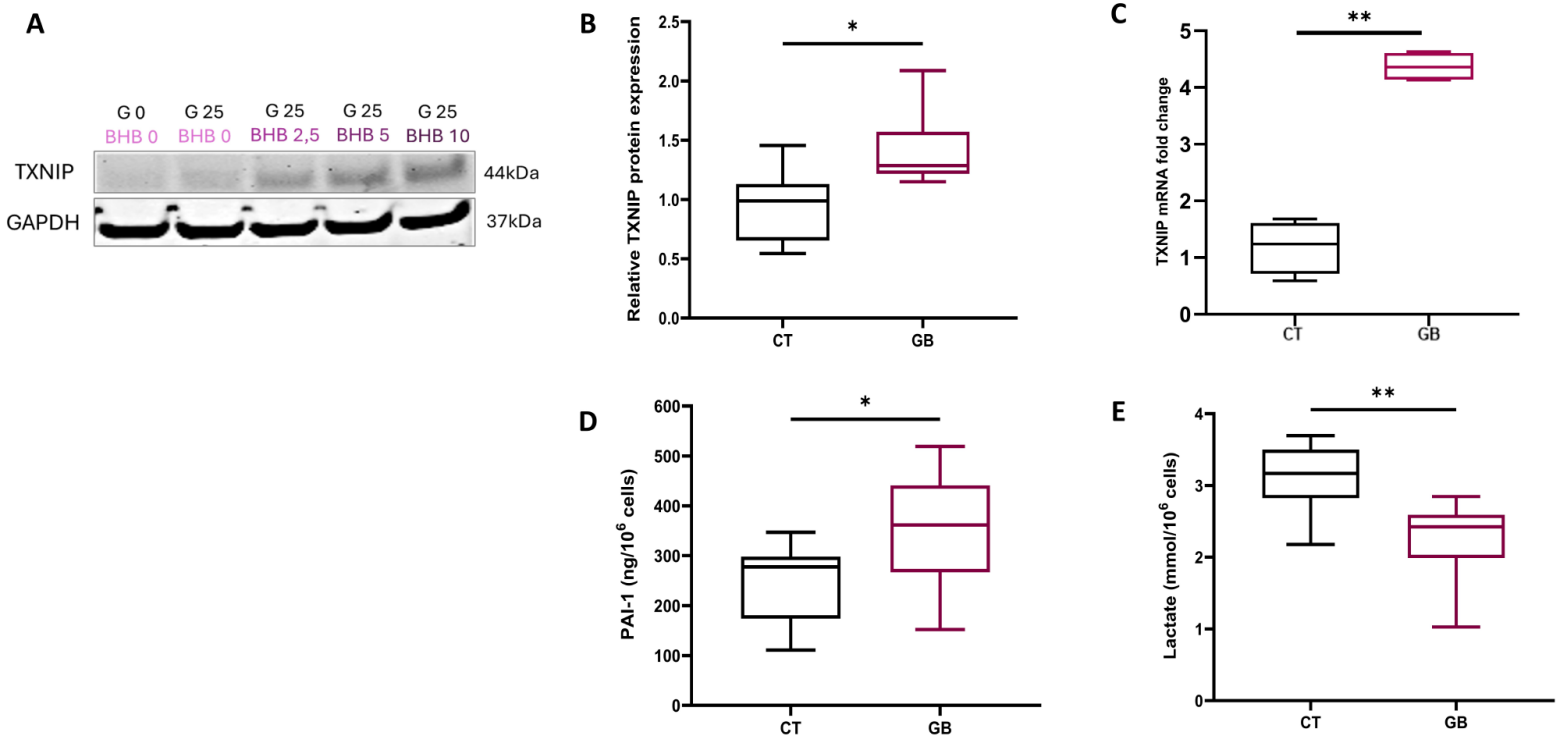
In vitro metabolic stress accurately simulates the age-related comorbidities observed in vivo. Experiments were carried out in ECFCs in control (CT) or metabolic stress conditions (GB). **A** Western blot analysis of TXNIP protein expression and GAPDH over a range of β-hydroxybutyrate levels in cell lysates of ECFCs. A representative cropped blot is shown (full-length blots are presented in Additional file 2: Figure S2) **B** Protein expression of TXNIP measured by Western blot (*n* = 6) **C** TXNIP mRNA expression assessed by RTqPCR (*n* = 4) **D** Quantification of PAI-1 secretion (ng/10^6^ cells) in ECFC conditioned media assessed by Luminex assay (*n* = 6) **E** Concentration of lactates (mmol/L) (*n* = 5). *p < 0,05; **p < 0,01

To further elucidate the role of age-related comorbidities and unravel the mechanisms behind their adverse effects, we refined our study on the role of endothelial progenitors based on previous findings [23]. Our in vivo model of age-related comorbidities revealed increased β-hydroxybutyrate and decreased lactate levels (see Additional file 1: Figure S1). To mimic these comorbidities, we established an in vitro metabolic stress model by enriching ECFC culture medium with glucose and β-hydroxybutyrate. After testing at increasing concentrations (Fig. 1A), 25 mM glucose and 10 mM β-hydroxybutyrate (GB) were added to replicate the in vivo cellular environment.

Interestingly, changes were observed in TXNIP expression and PAI-1 secretion. Mimicking age-related comorbidities with metabolic stress resulted in increased TXNIP protein and mRNA expression (+ 1.4-fold, *p* < 0.05; and + 4.4-fold, *p* < 0.01, respectively) (Fig. 1B, C) and PAI-1 secretion (+ 41%, *p* < 0.05) (Fig. 1D) and in decreased lactate levels (-24%, *p* < 0,01) (Fig. 1E) in ECFCs.

### TXNIP expression and silencing: revealing the impact of TXNIP on age-related comorbidities in ECFCs

**Fig. 2:**
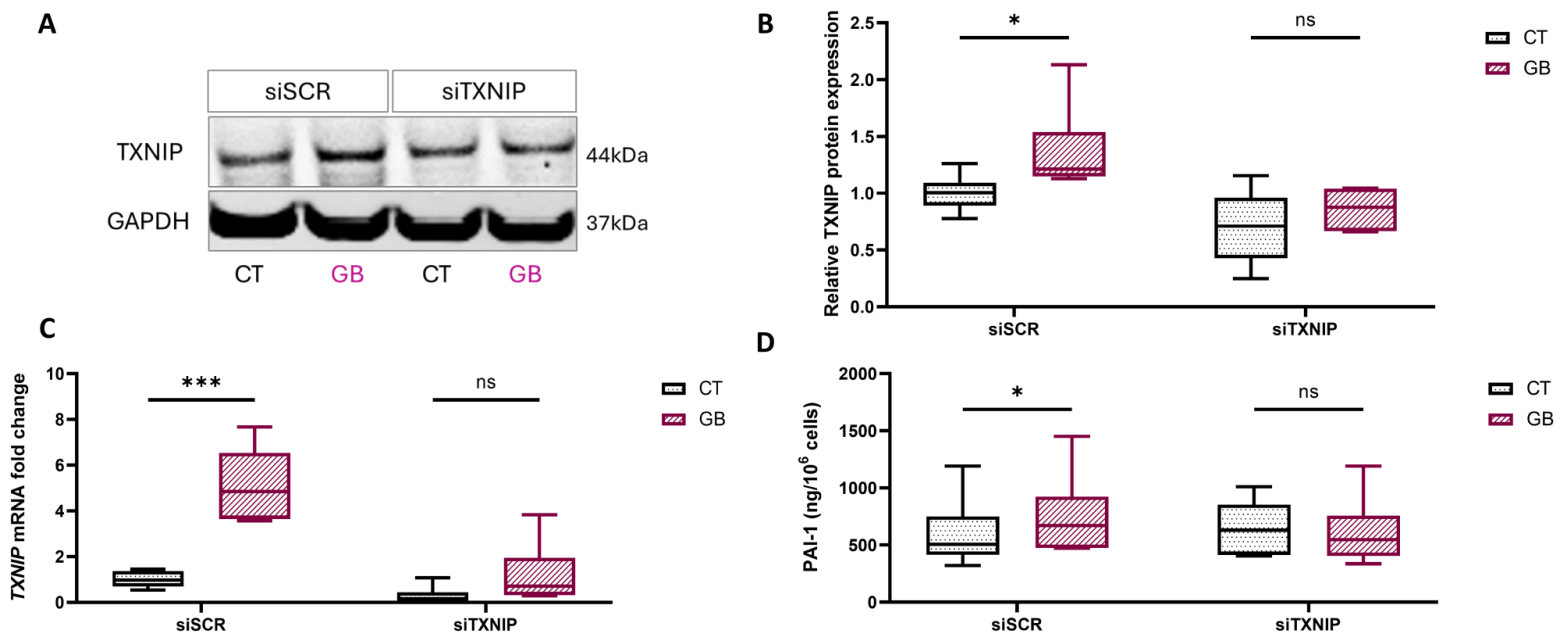
TXNIP expression and silencing: revealing the impact of TXNIP on age-related comorbidities in ECFCs. Experiments were carried out in ECFCs in transfected control cells with (siSCR GB) or without (siSCR CT) metabolic stress conditions and transfected siRNA TXNIP cells with (siTXNIP GB) or without (siTXNIP CT) metabolic stress conditions. Each media group was compared pairwise. **A**,** B** Western blot analysis of TXNIP protein expression and GAPDH in cell lysates of ECFCs (*n* = 6). A representative cropped blot is shown (full-length blots are presented in Additional file 2: Figure S2) **C** Quantification of TXNIP mRNA expression by RTqPCR (*n* = 6) **D** Quantification of PAI-1 (ng/10^6^ cells) in ECFC conditioned media assessed by Luminex assay. Effect of metabolic stress and TXNIP silencing on PAI-1 secretion (*n* = 6). *p < 0,05; ***p < 0,001; ns: not significant

To elucidate the role of TXNIP in our in vitro model of metabolic stress that simulates age-related comorbidities, we employed a transfection method. This method allows us to investigate the function of TXNIP by dissecting its contribution to cellular dysfunction and to counteract TXNIP overexpression triggered by the in vitro metabolic stress model mimicking age-related comorbidities. In the transfection control, we observed an increase in TXNIP at both the protein and mRNA levels (+ 1.3-fold, *p* < 0.05; and + 5-fold, *p* < 0.001, respectively) (Fig. 2A-C) induced by metabolic stress conditions simulating age-related comorbidities. Importantly, TXNIP silencing prevented the stress-induced increase, effectively blocking the upregulation of TXNIP protein expression (Fig. 2A, B) and TXNIP mRNA expression (Fig. 2C). This outcome demonstrated successful mitigation of metabolic stress through transfection. The effects of TXNIP silencing are also reflected in the secretion capacity of ECFCs, as metabolic stress induces increased secretion of PAI-1 (+ 27%, *p* < 0,05), which is mitigated by TXNIP silencing, resulting in reduced PAI-1 secretion (Fig. 2D).

### Impact of TXNIP silencing on vasculogenic properties and senescence under metabolic stress

**Fig. 3:**
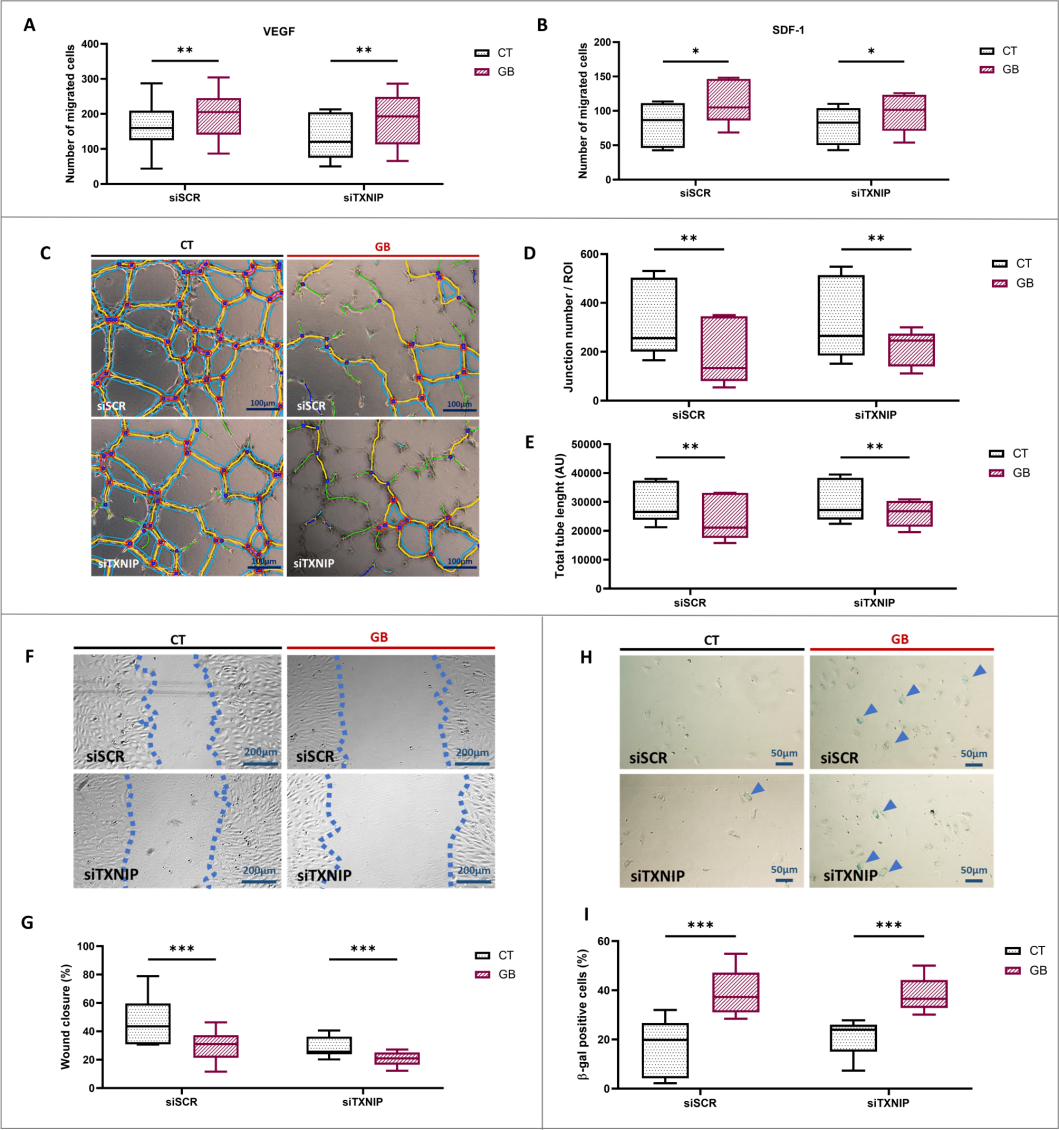
Impact of TXNIP silencing on vasculogenic properties and senescence under metabolic stress. Experiments were carried out in ECFCs in transfected control cells with (siSCR GB) or without (siSCR CT) metabolic stress conditions and transfected siRNA TXNIP cells with (siTXNIP GB) or without (siTXNIP CT) metabolic stress conditions. Each group was compared pairwise. Chemotactic migration assay in Boyden chamber with **A** VEGF or **B** SDF-1, number of migrated cells (*n* = 5–6). **C** Tubulogenesis in Matrigel™ model: representative images of vascular network at T4h (x40 magnification) and **D** quantification of junction number formed and **E** total length of tubes (AU) (*n* = 5) **F** Wound healing assay: representative images of the wound at T4h (x40 magnification) and **G** quantification of percentage of wound closure (*n* = 6–7). **H** Senescence assay: images of senescent cells (in blue) (x200 magnification) and **I** quantification of percentage of positive cells to β-galactosidase staining (*n* = 5). *p < 0,05; **p < 0,01; ***p < 0,001

In the presence of VEGF or SDF-1, which are chemoattractant cytokines, metabolic stress increased ECFC migration across the transwell (respectively, + 28%, *p* < 0,01; and + 48%, *p* < 0,05) (Fig. 3A, B). Similarly, ECFC migration was enhanced in the presence of metabolic stress in cells transfected with siTXNIP in the presence of VEGF or SDF-1 (+ 43%, *p* < 0,01; +27%, *p* < 0,05, respectively).

Our model of metabolic stress that simulates age-related comorbidities led to a reduction in tubulogenesis, evidenced by decreased junction number and network length in the siSCR groups (-48%, *p* < 0,01; and − 19%, *p* < 0,01, respectively) (Fig. 3C-E). This reduction was also observed in the siTXNIP GB group compared to the siTXNIP CT group (-32%, *p* < 0,01; and − 13%, *p* < 0,01, respectively).

In the wound healing assay, the percentage of wound closure decreased under metabolic stress conditions with and without TXNIP silencing (-30%, *p* < 0,001; and − 38%, *p* < 0,001, respectively) (Fig. 3F, G).

Metabolic stress increases cellular senescence, indicated by a rise in β-galactosidase-positive cells (+ 233%, *p* < 0,001), and TXNIP silencing does not protect against this increase (siTXNIP GB + 95%, *p* < 0,001 vs. siTXNIP CT) (Fig. 3H, I).

### Impact of TXNIP silencing on IL-8 secretion by ECFCs and neutrophil recruitment under metabolic stress

Following the examination of the vasculogenic properties of ECFCs, we shifted our focus to assess how their secretory function might be altered under metabolic stress conditions simulating age-related comorbidities. Since endothelial TXNIP deletion exhibits anti-inflammatory and proangiogenic effects in vivo [23, 27], we aimed to understand the effects of TXNIP silencing on the secretion of angiogenesis- and inflammation-related cytokines in ECFC conditioned media.

**Fig. 4:**
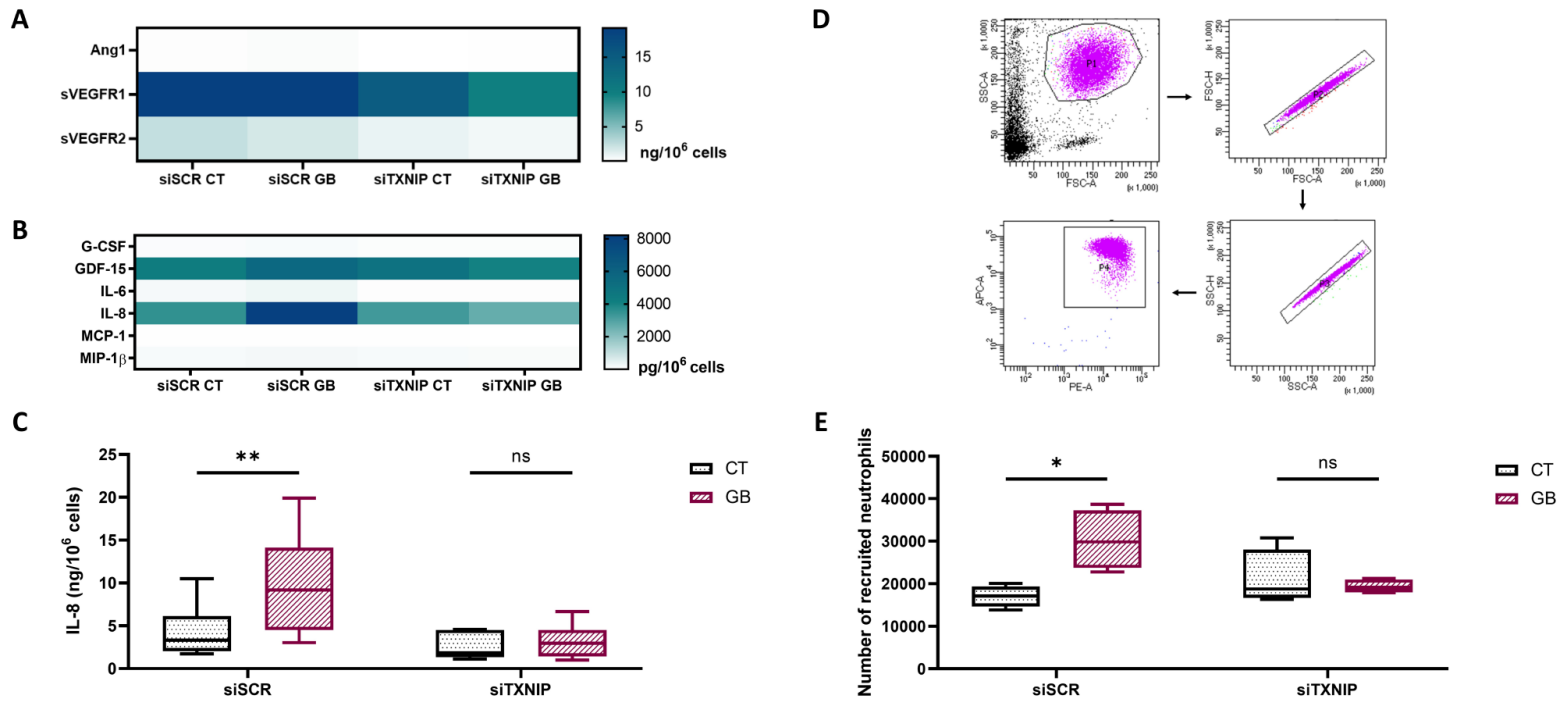
Impact of TXNIP silencing on IL-8 secretion by ECFCs and neutrophil recruitment under metabolic stress. Experiments were carried out in ECFCs in transfected control cells with (siSCR GB) or without (siSCR CT) metabolic stress conditions and transfected siRNA TXNIP cells with (siTXNIP GB) or without (siTXNIP CT) metabolic stress conditions. Each media group was compared pairwise. Quantification of the various cytokines in conditioned media was assessed by Luminex assay. **A** Quantification of the pro-angiogenic cytokine panel (ng/ 10^6^ cells) (*n* = 6) **B** Quantification of the SASP cytokine panel (pg/10^6^ cells) The corresponding values to the heatmaps are detailed in Additional file 3: Table S3. **C** Quantification of IL-8 (ng/ 10^6^ cells) (*n* = 6) **D** Gating strategy to select the purified neutrophils (PE-A for CD15 and APC-A for CD16) **E** Number of recruited neutrophils to ECFC conditioned media (*n* = 4). *p < 0,05; **p < 0,01; ns: not significant

Initially, we focused on angiogenic factors with the following panel: angiopoietin-1, PDGF-BB, sVEGFR-1 and − 2 and TGF-β. Some cytokines were not detectable by the kit, and others showed no difference between the groups (Fig. 4A). We then focused on the secretion of inflammatory and senescence factors known as the senescence-associated secretory phenotype (SASP): G-CSF, GDF-15, IL-1β, IL-6, IL-8, MCP-1, MIP-1α, MIP-1β, MMP-9, RANTES and TNF-α. As before, the concentrations of some cytokines were below the detection kit limits. As shown in Fig. 4B, G-CSF, IL-6, MCP-1 and MIP-1β were secreted at very low levels and in a comparable manner regardless of the group. The cytokines GDF-15 and IL-8 are abundantly secreted, but IL-8 is the most relevant cytokine since its levels vary significantly between groups (see detailed values in Additional file 3: Table S3). Indeed, metabolic stress let to a significant increase in IL-8 secretion (+ 132%, *p* < 0,01) (Fig. 4C), which was not observed with TXNIP silencing.

Considering these findings on IL-8, we further investigated neutrophil recruitment using a transwell migration assay. As shown in Fig. 4D and E, metabolic stress significantly increased the percentage of neutrophils recruited (+ 76%, *p* < 0,05). Importantly, TXNIP silencing prevented the increase in neutrophil recruitment induced by metabolic stress In vitro.

### Endothelial TXNIP deletion prevents MIP-2 secretion, leading to neutrophil recruitment induced by age-related comorbidities

Twenty-one days after hindlimb ischemia, we collected serum for the MIP-2 assay and calf muscle sections for MPO staining. Indeed, inflammation and neutrophil recruitment have been studied. We assayed an IL-8 homolog in mice, MIP-2, and measured the number of MPO positive cells.

**Fig. 5:**
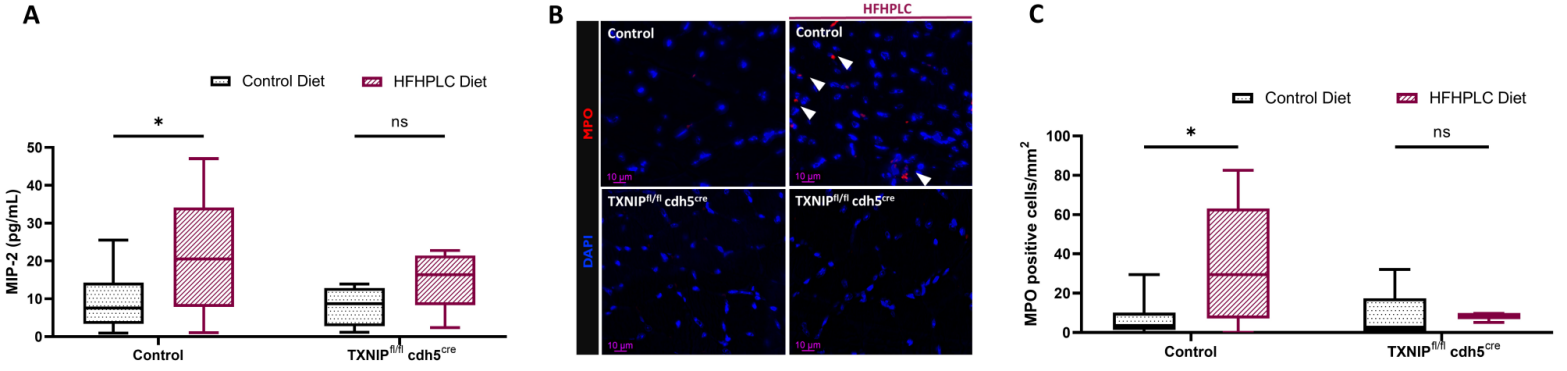
Endothelial TXNIP deletion prevents MIP-2 secretion, leading to neutrophil recruitment induced by age-related comorbidities. Experiments were carried out in 4 groups: (i) cre-cadherin littermate control mice fed a standard control diet (control with control diet); (ii) cre-cadherin littermate control mice fed an HFHPLC diet (control with HFHPLC diet); (iii) TXNIP^fl./fl.^cdh5^cre^ fed a standard control diet (TXNIP^fl./fl.^cdh5^cre^ with control diet); and (iv) TXNIP^fl./fl.^cdh5^cre^ fed an HFHPLC diet (TXNIP^fl./fl.^cdh5^cre^ with HFHPLC diet). Each dietary group was compared pairwise. Samples were taken from mice 21 days after hindlimb ischemia. **A** MIP-2 concentration (pg/ml) in mice serum assessed by ELISA (control mice *n* = 10–15; TXNIP^fl.^/^fl.^cdh5^cre^ mice *n* = 4–5) **B** Representative images of positive cells to MPO staining on calf muscle (x40 magnification) **C** Quantification of MPO positive cells (MPO positive cells/mm²) (control mice *n* = 7–10; TXNIP^fl.^/^fl.^cdh5^cre^ mice *n* = 3–5). *p < 0,05; ns: not significant

In WT mice, compared with the control diet, the high-fat high-protein low carbohydrate diet (HFHPLC diet) significantly increased the serum MIP-2 concentration (+ 126%, *p* < 0,05) (Fig. 5A). However, in TXNIP^fl/fl^cdh5^cre^ mice, the HFHPLC diet had no effect in MIP-2 levels.

As depicted in Fig. 5B and C, the HFHPLC diet led to a substantial increase in the number of MPO positive cells compared with the control diet (+ 576%, *p* < 0,05). Conversely, in TXNIP^fl/fl^cdh^5cre^ mice, the HFHPLC diet did not affect the number of MPO positive cells in the muscle.

## Discussion

Significant attention has been given to the role of TXNIP in the context of aging [32, 33], diabetes or hyperglycemia [34–36] and age-related comorbidities [27]. Endothelial TXNIP contributes to arterial wall inflammation, smooth muscle cell loss and contractile dysfunction [27, 36] and has been further explored in the context of angiogenesis and postischemic revascularization in a hindlimb ischemia model of age-related comorbidities [23]. In this study, we employed two approaches to target endothelial TXNIP: a transgenic method and a systemic siRNA injection technique. Targeting endothelial TXNIP successfully restored impaired blood flow, rescued the impaired cell response to proangiogenic factors, reduced necrosis scores, attenuated muscle damage and decreased the infiltration of immune cells and adipocytes induced by age-related comorbidities by day 21 [23]. Although both the transgenic and systemic siRNA methods confirmed that targeting endothelial TXNIP enhances revascularization, the effects were more pronounced in the transgenic model. This finding suggested that while silencing TXNIP in mature endothelial cells is beneficial, targeting endothelial progenitors could significantly enhance revascularization outcomes. This finding aligns with the hypothesis previously described in the literature that the Cre–lox method affects both mature endothelial cells and endothelial progenitors [31]. However, there has been a lack of research on the specific implications of TXNIP in endothelial progenitors.

To further explore the beneficial effects of TXNIP silencing on revascularization outcomes, we undertook an in vitro investigation focused on endothelial progenitors, specifically ECFCs. To mimic age-related comorbidities observed in vivo and to induce metabolic stress in vitro, we added 25 mM glucose and 10 mM β-hydroxybutyrate to the culture medium. This choice was based on the observed metabolic dysregulation in mice with age-related comorbidities, notably characterized by elevated β-hydroxybutyrate levels. Additionally, these mice exhibited reduced lactate levels, a reduction also observed in vitro, confirming a metabolic shift in endothelial cells toward reduced glycolysis. Moreover, specific concentrations of glucose and β-hydroxybutyrate were chosen because they effectively increased TXNIP protein expression and PAI-1 secretion in our experiments, thus accurately reflecting in *vivo* endothelial conditions [27]. In the literature, in vitro models typically focus on simulating either hyperglycemia or hyperlipidemia, but rarely attempt to reproduce the comprehensive metabolic context of age-related comorbidities [37–39]. This represents a significant limitation, as it does not fully capture the multifactorial nature of aging in vivo along with its comorbidities. Our experimental model addresses this gap by offering a more holistic view of metabolic disorders.

Ultimately, our metabolic stress model induces TXNIP overexpression at both the RNA and protein levels in ECFCs, similar to the effects of hyperglycemic stress observed in mice circulating EPCs [40]. To counter this metabolic stress-induced overexpression, we employed RNA silencing technology, which strongly reduced the PAI-1 secretion induced by metabolic stress. This finding highlights the effectiveness of TXNIP silencing, not only in reducing TXNIP levels but also in mitigating downstream paracrine effects such as PAI-1 secretion, a key factor in the pathological response to metabolic stress [27, 41]. Although no specific biosafety studies on progenitor modification for clinical translation were conducted, we monitored TXNIP expression dynamics post-silencing, revealing a transient effect lasting up to 72 h post-transfection (data not shown), sufficient to impact repair and highlighting siRNA’s short lifespan.

Postischemic EPC recruitment is driven by chemokines such as VEGF and SDF-1, which enhance their release and mobilization. Using a Boyden chamber assay, we observed that metabolic stress promoted ECFC recruitment in response to chemoattractant stimuli such as VEGF or SDF-1, regardless of TXNIP silencing. We also investigated the vasculogenic properties of ECFCs under metabolic stress, both with and without TXNIP silencing. Metabolic stress reduced both tubulogenesis and migration in ECFCs, findings that are consistent with those observed in ECFCs exposed to high glucose levels [37–39] and in ECFCs from patients with diabetes or severe atherosclerotic cardiovascular disease [42, 43]. However, in our study, TXNIP silencing did not mitigate these effects. Resveratrol, a pharmacological inhibitor of TXNIP, has been shown to modify tube formation in EPCs, with effects varying with dose. Similarly, it modulates cellular senescence [44–46]. Senescence has been observed in EPCs isolated from diabetic patients [46, 47]. In our experiments, TXNIP inhibition did not affect metabolic stress-induced senescence in ECFCs, as measured by the activity of SA-β-galactosidase, a common biomarker of senescent cells [48]. Our findings showed that TXNIP does not counter the metabolic stress-induced increase in migration or impairment of the vasculogenic properties of the ECFC. Moreover, we showed that ECFCs became senescent under these conditions, with no mitigating effects from TXNIP silencing. This suggests that the inability of TXNIP to modify senescence contributes to the observed reductions in tubulogenesis and migration capabilities under stress conditions.

Given our previous findings on the role of endothelial TXNIP in the paracrine secretion of proinflammatory and prothrombotic factors [23, 27], we focused on the secretory behaviors of ECFCs. After observing no changes in proangiogenic factors, we specifically examined their ability to release inflammatory cytokines and factors associated with the “senescence-associated secretory phenotype” (SASP). The detection threshold of the multiplex kit limited our analysis; however, our secretome analysis indicated that GDF-15 and IL-8 were abundantly secreted, with a notable increase in IL-8 under metabolic stress. Importantly, the paracrine effects of stressed ECFCs are significant because ECFCs do not express the IL-8 receptors CXCR1 and CXCR2 [49]. Previous studies have linked proinflammatory and prosenescent IL-8 secretion [50–52], and concentration gradients of IL-8 are known to facilitate neutrophil recruitment and regulate transendothelial neutrophil migration [53, 54]. Moreover, IL-8 secretion by senescent ECFCs has even been associated with increased neutrophil migration [52]. Previous studies have reported that a pharmacological inhibitor of TXNIP, resveratrol, reduces IL-8 secretion in ECFCs [55]. Interestingly, our results demonstrated that TXNIP silencing in ECFCs decreases metabolic stress-induced IL-8 secretion and significantly impairs metabolic stress-induced neutrophil migration.

As our previous study demonstrated that endothelial TXNIP deletion reduces the number of infiltrating cells in ischemic muscle after 21 days in our mouse model of age-related comorbidities [23], our current study confirms that endothelial TXNIP deletion also protects against increased neutrophil infiltration. The extent of neutrophil infiltration was quantitatively assessed using myeloperoxidase. Although we acknowledge that the small sample sizes in the transgenic models of endothelial TXNIP deletion might reduce the statistical robustness of our findings, the consistent observation of decreased neutrophil infiltration across various experiments supports the validity of our results [23]. This consistency with our previous results suggests a strong and replicable biological effect of endothelial TXNIP deletion. Reducing neutrophil infiltration is a recognized therapeutic strategy for enhancing revascularization in ischemia, as reported in diabetes-related studies [56]. Endothelial TXNIP deletion also appears to protect against increased secretion of MIP-2, the mouse equivalent of IL-8. MIP-2 is crucial for chemotaxis at inflammatory sites and neutrophil activation. This protective role of TXNIP in targeting elevated MIP-2 levels suggests its potential for moderating inflammatory responses exacerbated by age-related comorbidities [57]. In the literature, genetic targeting of murine CXCR2 linked to MIP2 has been shown to inhibit neutrophil infiltration of inflamed tissue [58]. Although a persistent increase in MIP-2 levels in muscle 24 h postreperfusion was not associated with increased edema or injury [59], the persistent increase after 21 days appears to be due to the age-related comorbidities. Indeed, without comorbidities, the number of neutrophils infiltrating ischemic muscle increases rapidly and then completely disappears by day 7 postischemia or day 14 according to the kinetics of various studies [60–62]. Neutrophils play dual roles in angiogenesis; depending on the environment and stage of the immune response, they can promote angiogenesis [63–65] or inhibit angiogenesis by releasing enzymes and reactive oxygen species that degrade angiogenic factors and damage tissue [12]. We conclude that excessive accumulation and/or delayed removal of neutrophils are deleterious. Interestingly, endothelial TXNIP deletion, such as TXNIP silencing in ECFCs, can reduce neutrophil inflammation in a deleterious context.

Our key findings are as follows:

(i) TXNIP silencing did not affect increased ECFC recruitment or metabolic stress-induced decreases in tubulogenesis, migration, or senescence.
(ii) TXNIP silencing significantly decreases metabolic stress-induced IL-8 secretion, which directly impacts neutrophil recruitment under these conditions.
(iii) Endothelial TXNIP deletion effectively prevents MIP-2 secretion, subsequently reducing age-related comorbidities-induced neutrophil recruitment in our in vivo model.

Our study demonstrated that TXNIP silencing plays a crucial role in modulating the inflammatory response in ECFCs under metabolic stress, specifically through its impact on IL-8 secretion and subsequent neutrophil recruitment. This study not only highlights the significance of TXNIP in reducing inflammatory responses but also emphasizes the need for further investigations to delineate the specific molecular pathways and cellular interactions it modulates. Understanding these mechanisms will enhance our knowledge of TXNIP’s comprehensive role in inflammatory processes and its potential therapeutic implications in conditions of metabolic stress. These findings underscore the complexity of TXNIP’s function and pave the way for future research aimed at exploiting its therapeutic potential to translate these findings into clinical practice. To advance ECFCs for clinical development, there are several challenges that need to be addressed. Targeting TXNIP may contribute to addressing issues related to heterogeneity in disease states [66–68].

## Conclusions

In conclusion, targeting endothelial TXNIP in ECFCs mitigates the adverse effects of metabolic stress, such as an impaired response to ischemia, by modulating the secretion of proinflammatory factors, mainly IL-8, and influencing neutrophil dynamics. This protective mechanism of targeting TXNIP has been validated in a mouse model of age-related comorbidities, revealing significant advances in understanding the cell-specific responses of endothelial lineage TXNIP to metabolic stress. Importantly, this study is the first to demonstrate that endothelial TXNIP has a cell-specific effect on regulating infiltration and immune cell damage under metabolic stress. Although targeting TXNIP in endothelial progenitors alone may not sufficiently enhance their therapeutic capabilities in cell therapies, it is crucial for protecting these cells from the harmful environments typically found in patients with age-related comorbidities. This insight could pave the way for targeting TXNIP in ECFCs may alleviate ischemic complications exacerbated by metabolic stress, offering potential clinical benefits for patients suffering from age-related comorbidities.

## Electronic supplementary material

Below is the link to the electronic supplementary material.

Supplementary Material 1

Supplementary Material 2

Supplementary Material 3

## Data Availability

The raw datasets generated and/or analyzed during the current study will be made available upon request to the corresponding author.

## Abbreviations

Ang1: Angiopoietin-1
BHB: β-hydroxybutyrate
CT: Control medium
CXCR-1: CXC motif chemokine receptor-1
CXCR-2: CXC motif chemokine receptor-2
ECFCs: Endothelial colony-forming cells
EPCs: Endothelial progenitor cells
GAPDH: Glyceraldehyde-3-phosphate dehydrogenase
GB: Glucose and β-hydroxybutyrate supplemented medium
G-CSF: Granulocyte-colony stimulating factor
GDF-15: Growth differentiation factor-15
HFHPLC: High-fat high-protein low carbohydrate
MACs: Myeloid angiogenic cells
MCP-1: Monocyte chemoattractant protein-1
MIP-1α: Macrophage inflammatory protein-1α
MIP-1β: Macrophage inflammatory protein-1β
MIP-2: Macrophage inflammatory protein-2
MMP-9: Matrix metalloproteinase-9
MPO: Myeloperoxidase
PAI-1: Plasminogen activator inhibitor-1
PDGF-BB: Platelet derived growth factor-BB
RANTES: Regulated upon activation, normal T cell expressed and secreted
SA- β-gal: Senescence-associated ß-galactosidase
SDF-1: Stromal-derived factor-1
sVEGFR-1: Soluble vascular endothelial growth factor receptor-1
sVEGFR2: Soluble vascular endothelial growth factor receptor-2
TGF-β: Transforming Growth Factor-β
TNF-α: Tumor necrosis factor-α
TXNIP: Thioredoxin-interacting protein
VEGF: Vascular endothelial growth factor
