# Supplementary figures and images for: Targeting TXNIP in endothelial progenitors mitigates IL-8-induced neutrophil recruitment under metabolic stress
Source: Stem Cell Res Ther. 2024 Jul 29;15:225. doi: 10.1186/s13287-024-03850-w (PMC11287885; doi:10.1186/s13287-024-03850-w)

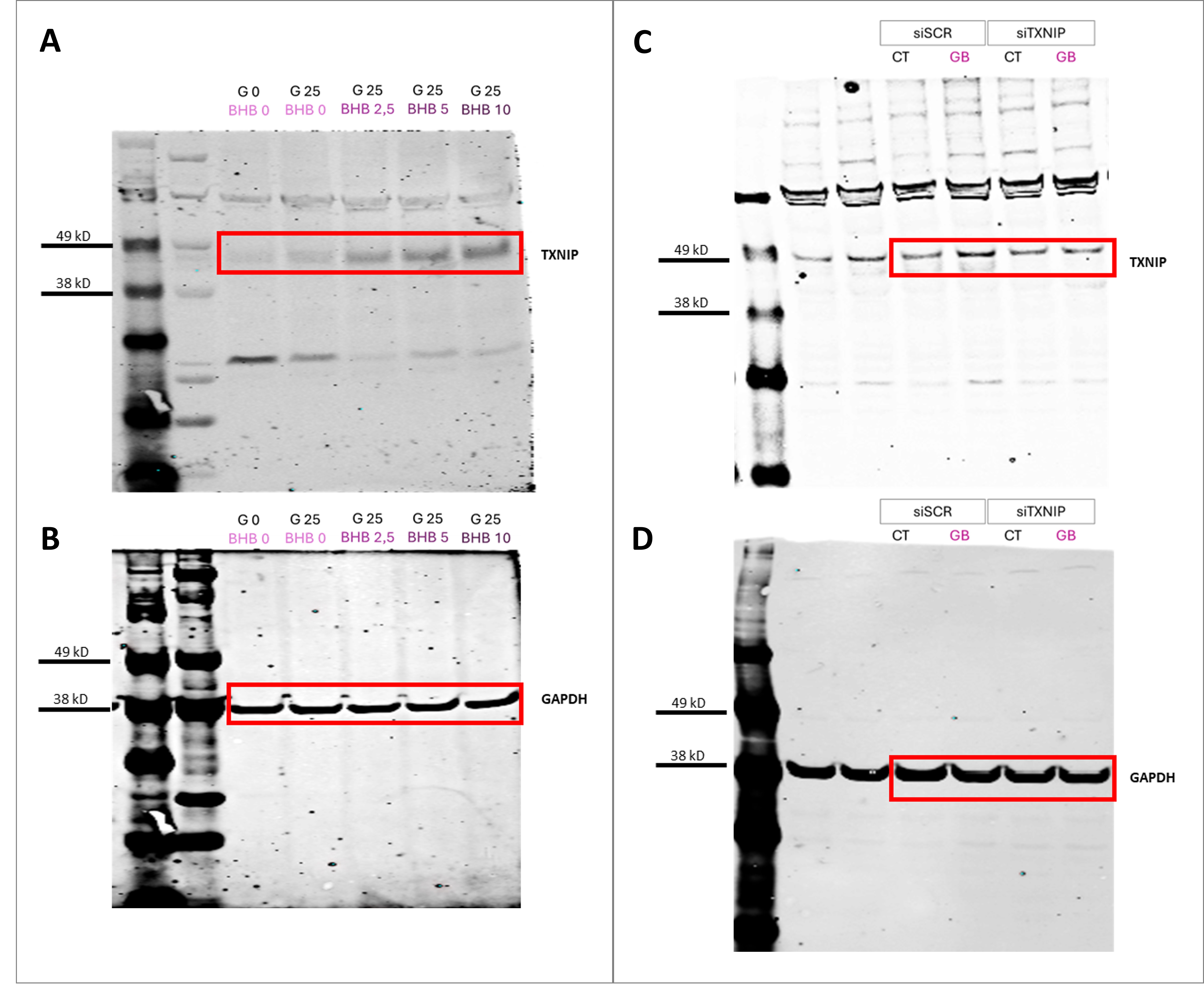

Supplement: Supplementary file 1 — Supplementary Material 1 [file 13287_2024_3850_MOESM1_ESM.jpg]

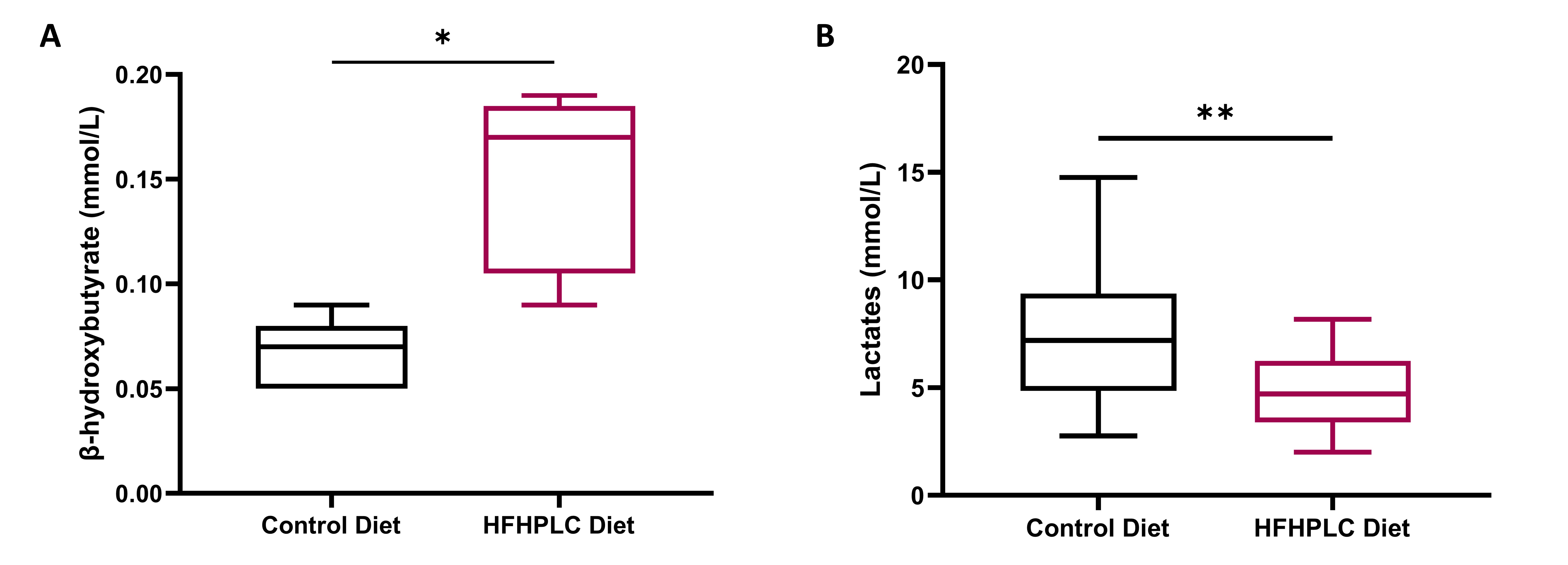

Supplement: Supplementary file 2 — Supplementary Material 2 [file 13287_2024_3850_MOESM2_ESM.jpg]

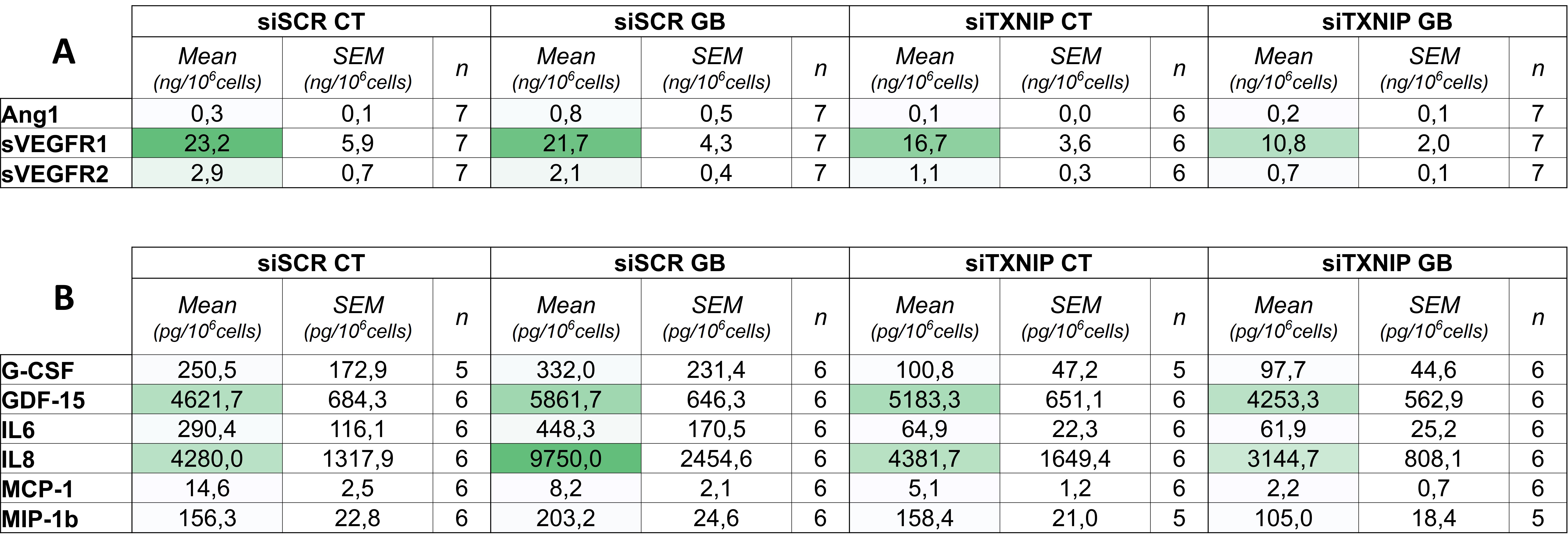

Supplement: Supplementary file 3 — Supplementary Material 3 [file 13287_2024_3850_MOESM3_ESM.jpg]
